# Sea-level feedback lowers projections of future Antarctic Ice-Sheet mass loss

**DOI:** 10.1038/ncomms9798

**Published:** 2015-11-10

**Authors:** Natalya Gomez, David Pollard, David Holland

**Affiliations:** 1 Center for Atmosphere Ocean Sciences, Couratnt Institute of Mathematical Sciences, New York University, New York, New York 10012, USA; 2 Department of Earth and Planetary Sciences, McGill University, 3450 University Street, Montreal, Quebec, Canada H3A 0E8; 3 Earth and Environmental Systems Institute, Pennsylvania State University, University Park, Pennsylvania 16801, USA

## Abstract

The stability of marine sectors of the Antarctic Ice Sheet (AIS) in a warming climate has been identified as the largest source of uncertainty in projections of future sea-level rise. Sea-level fall near the grounding line of a retreating marine ice sheet has a stabilizing influence on the ice sheets, and previous studies have established the importance of this feedback on ice age AIS evolution. Here we use a coupled ice sheet–sea-level model to investigate the impact of the feedback mechanism on future AIS retreat over centennial and millennial timescales for a range of emission scenarios. We show that the combination of bedrock uplift and sea-surface drop associated with ice-sheet retreat significantly reduces AIS mass loss relative to a simulation without these effects included. Sensitivity analyses show that the stabilization tends to be greatest for lower emission scenarios and Earth models characterized by a thin elastic lithosphere and low-viscosity upper mantle, as is the case for West Antarctica.

## Abstract (web summary)

The retreat of the Antarctic Ice Sheet and its potential contribution to future sea-level rise, is a major focus of climate research. Here, the authors show that positive feedbacks involving bedrock uplift and sea surface drop, may significantly impact the timing and extent of local ice-sheet retreat.

## Graphical abstract

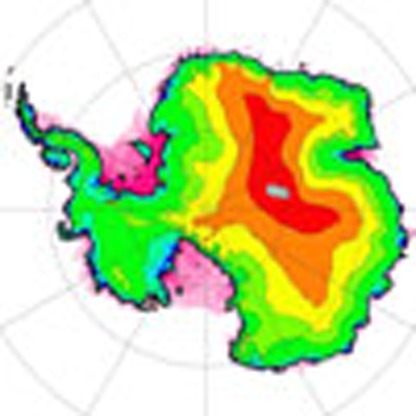

The Antarctic Ice Sheet (AIS) has the potential to make a significant contribution to future sea-level rise^1^. Large-scale collapse of some marine sectors of the AIS may already be underway^2^,^3^,^4^,^5^, but the timing and extent of future retreat is uncertain. Marine-based sectors of the AIS gain most of their mass through precipitation and lose mass by ice outflux at the grounding line^6^. Ice-sheet mass loss leads to a sea-level (that is, water depth) fall at the grounding line, both because of post-glacial rebound of the unloaded crust and a drop in sea surface height as water migrates away from the ice sheet because of reduced gravitational attraction^7^,^8^. Ice flux is highly sensitive to ice thickness at the grounding line of a marine ice sheet^6^, which is in turn proportional to the depth of water there. Thus, the aforementioned sea-level fall reduces the ice outflux at the grounding line and acts as a stabilizing influence on the ice sheet^9^. Previous studies of the effect have focused on the long-term, ice age evolution of the AIS (and other ice sheets) using coupled ice sheet–sea-level models of varying complexity (for example, see refs 10, 11, 12, 13 for details), and all have verified the universality of the self-stabilizing mechanism.

Here we apply the most advanced type of these models^11^,^14^ to explore, for the first time, the impact of the sea-level feedback on projections of AIS collapse under a wide range of future emission scenarios. We also adopt several models of the Earth's viscoelastic structure in the sea-level modelling to capture the range of gravitational, rotational and bedrock deformational responses to the ice-ocean mass redistribution. We note that long timescale, large spatial scale ice-sheet modelling studies include treatments of bedrock deformation (for example, see refs 14, 15, 16, 17 for details), but recent regional studies of future AIS retreat over up to hundreds of years have kept the bedrock elevation fixed (for example, see refs 2, 5 for details), and none of these studies have included gravitational effects on sea level. Our results indicate that sea-level changes associated with gravitational effects and deformation of the solid Earth may impact future Antarctic ice-sheet evolution. Moreover, the adopted viscoelastic Earth structure influences the size and timing of the impact of the sea-level feedback.

## Results

### Impact of sea-level changes on future ice loss

We use a coupled ice sheet–sea-level model^11^ to simulate AIS thickness and bedrock evolution and global sea-level changes under a series of climate forcing scenarios derived from Regional Climate Model (RCM) simulations (see Methods for details).

**Figure 1:**
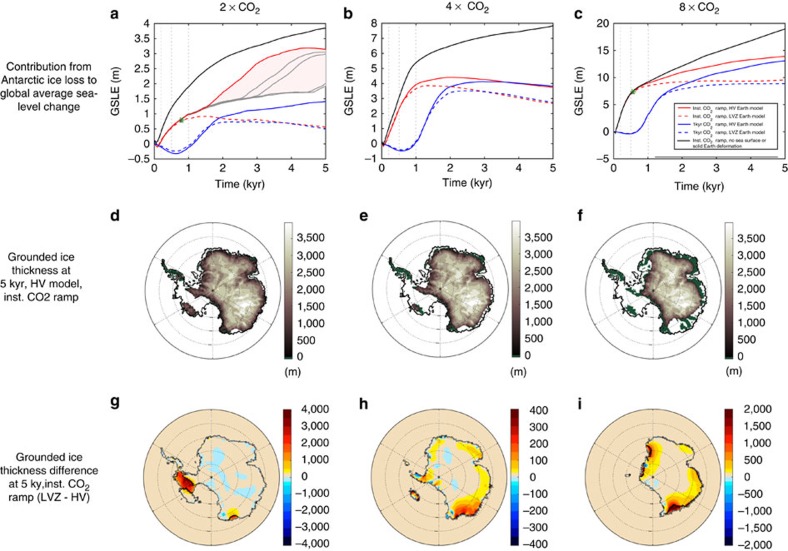
Impact of sea-level changes on AIS evolution for a suite of greenhouse emission scenarios. Results are shown for CO_2_ levels of 2 (left column), 4 (middle column) and 8 (right-most column) times modern levels. (**a**–**c**) Changes in grounded ice cover, in units of metres of GSLE, as a function of time for simulations in which CO_2_ levels are increased to the final value instantaneously (red and black lines) or in 1 kyr (blue lines). Projections based on the HV and LVZ Earth models are given by solid and dashed lines, respectively. Black lines correspond to a simulation in which the sea-level stabilization mechanism is not included. Grey lines within the pink shaded area of **a** show the results of simulations adopting the HV model and instantaneous CO_2_ ramp up, with a range of small changes to the ice-sheet model setup (see text). Green stars in **a**,**c** indicate the specific times considered in Fig. 2. Vertical grey-dashed lines highlight times (200 years, 500 years and 1 ky) discussed in the text. (**d**–**f**) Grounded ice thickness, in metres, for the instantaneous CO_2_ ramp up, HV Earth model simulations at 5 kyr. Green indicates region of exposed, positive bedrock topography at 5 ky, and black lines show the modern grounding line position. (**g**–**i**) Difference in grounded ice thickness in metres between the LVZ and HV Earth model simulations (solid red minus dashed red lines), with instantaneous CO_2_ ramp up, at 5 kyr, where the grey and black lines show the grounding line position of the former and latter simulations. Note the different colour bars on frames (**g**–**i**).

Coupled model simulations for RCM-based climate warming scenarios are shown in Fig. 1. In these simulations, CO_2_ levels in the atmosphere are linearly increased instantaneously (that is, over 1 year) or over 1 kyr (kilo-years; ‘gradual' forcing) at the start of the model run to two, four or eight times modern levels. This warming is applied by averaging together the appropriate RCM results for modern climatology and the various fixed, elevated atmospheric CO_2_ levels. Ocean temperatures are linearly, uniformly increased by 2 °C above modern climatology over the same time interval (see Methods). The black line in Fig. 1a–c is a control run, in which CO_2_ levels are increased instantaneously and no sea-level feedback is included; that is, the bedrock and sea surface height elevations in the ice-sheet model are fixed in time. The solid red and blue lines represent simulations that adopt an Earth model (henceforth model HV for ‘high viscosity') with lithospheric thickness of 120 km, and upper and lower mantle viscosities of 5 × 10^20^ and 5 × 10^21^ Pa s, respectively, in the sea-level calculations. This model is representative of a class of viscoelastic Earth models that satisfy a range of globally distributed ice age data sets (for example, see refs 18, 19 for details). All simulations are run for a total of 5 kyr.

The control run for the case of a 2 × CO_2_ emissions scenario is characterized by a loss of ∼2.8 × 10^6^ km^3^ of ice by the end of the simulation, which corresponds to a global average sea-level equivalent (GSLE; which we define at a given time as the globally averaged sea-level rise after all solid surface depressions, that is, negative topography, left behind by marine sectors freed of ice at that time in the model simulation, are filled with melt water) of 3.8 m (Fig. 1a, black line). About half of this GSLE rise (1.9 m) is reached after just 1 kyr. When the sea-level feedback is incorporated into the simulation, the sea-level rise is reduced by ∼50% after 200 years, 500 years and 1 kyr, and by ∼20%, to 3.1 m, after 5 kyr.

In the case of the 4 × CO_2_ emission scenario, over the first kyr, the coupled model (Fig. 1b—solid red line) predicts that nearly all of the West Antarctic Ice Sheet (WAIS) collapses (Fig. 1e) with some additional peripheral retreat of the East Antarctic Ice Sheet (EAIS) relative to the 2 × CO_2_ case (Fig. 1d). The incorporation of the sea-level feedback lowers the AIS contribution to predicted future sea-level rise by ∼30% after 200 and 500 years, 25% after 1 kyr, and by 50%, from 7.8 to 3.7 m GSLE, after 5 kyr (Fig. 1b, black versus solid red lines). These trends continue when considering the 8 × CO_2_ simulations, in which complete collapse of the WAIS, and extensive areas of the EAIS, occurs (Fig. 1f). In this case, the GSLE rise is 9 m after 1 kyr and 13.9 m after 5 kyr using the HV Earth model (Fig. 1c); these represent reductions of 0 and 25% relative to the sea-level rise computed in the control run. We conclude that local sea-level (bedrock and sea surface height) changes associated with ice-sheet collapse will have a significant impact on future AIS retreat in a warmer world on both centennial and millennial timescales.

**Figure 2:**
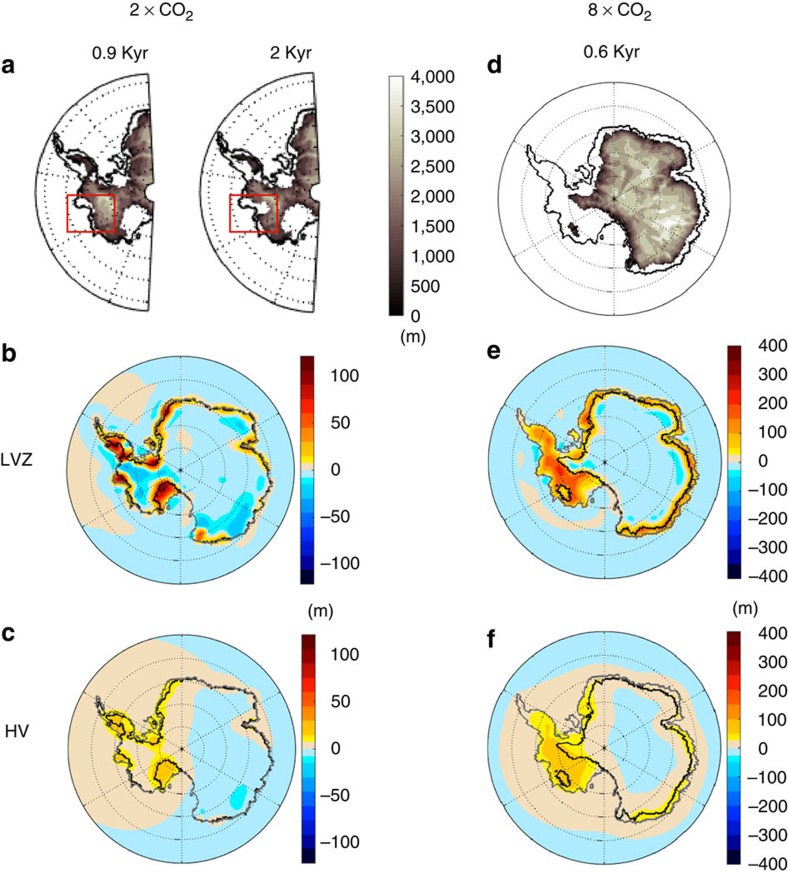
Ice-sheet retreat and bedrock elevation changes in simulations including the sea-level stabilization mechanism. Results for different viscoelastic Earth models and the 2 × CO_2_ (**a**–**c**) or 8 × CO_2_ (**d**–**f**) greenhouse emission scenarios. (**a**) Snapshots of grounded ice thickness in metres in West Antarctica for the HV Earth model simulation at 0.9 and 2 kyr. Black lines show the grounding line position at the start of the runs. The red box highlights the differences in the Amundsen Sea Sector. (**b**–**c**) Change in bedrock elevation, in metres, for simulations using the (**b**) LVZ and (**c**) HV Earth models from the start of the run to 0.9 kyr, when the ice distribution between the two simulations begins to diverge (see Fig. 1a). Black lines show the grounding line at 0.9 kyr, and grey lines show the grounding line at the start of the run (0 kyr). (**d**–**f**) Analogous to **a**–**c** for the 8 × CO_2_ emission scenario, except that ice thickness in **d** is shown over the whole AIS, and snapshots are shown at 0.6 kyr into the simulation.

In performing the above simulations, we noted interesting behaviour in the 2 × CO_2_ scenario for runs with the HV Earth model. In particular, the system reached an unsteady equilibrium state at ∼1.3 kyr whereby small changes to the ice-sheet model set up (for example, adopting time stepping in the ice-sheet model of between 0.075 and 0.25 years, which has negligible impact on other simulations) led to changes in timing of significant retreat in the Amundsen Sea Embayment (Fig. 1a; see also Fig. 2a). This retreat, equivalent to 1.5 m GLSE, occurred in all simulations with the 2 × CO_2_ emission scenario and the HV Earth model, but the onset of the retreat varied considerably (Fig. 1a). These results, which were not apparent for other emission scenarios or Earth models that we considered, imply that bedrock elevation and its evolution via sea-level change may be critical to grounding line migration in this region, and this suggests the need for future, high-resolution regional modelling and better constraints on bedrock conditions and elevation.

### Impact of Earth structure on future ice loss

Viscoelastic Earth structure beneath the AIS is highly variable. The EAIS is underlain by a stable, thick craton, and in contrast, a large continental rift system and thin lithosphere lie below the WAIS^20^. Moreover, seismic tomography indicates strongly variable and anomalously slow wave speeds in the shallow mantle below WAIS, suggesting a hot, low-viscosity asthenosphere^20^,^21^,^22^,^23^. It follows that the HV model, derived from a global distribution of ice age data sets, may not be appropriate to simulations of AIS stability, in particular in West Antarctica. To explore this issue, we repeated our simulations using an Earth model (henceforth LVZ for low-viscosity zone) that is characterized by an elastic lithosphere of 50 km thickness, a zone of relatively low viscosity (10^19^ Pa s) from the base of the lithosphere to 200 km depth, and viscosities of 2 × 10^20^ and 3 × 10^21^ Pa s in the rest of the upper mantle and in the lower mantle, respectively. (See Supplementary Fig.1 for results using a suite of different Earth models of which the HV and LVZ models are end members.)

Adopting the LVZ model increases the sea-level stabilization and reduces the predicted rise in GSLE for all emission scenarios as compared with the HV model, as viscous uplift takes place faster and deformation is more localized to the grounding line (compare the dashed and solid red lines in Fig. 1a–c, and ice distribution differences in Fig. 1g–i). After 5 kyr, the LVZ simulations show a GSLE rise that is 1.5–2.5 m less for the 2 × CO_2_ emission scenario (where the upper bound of this range reflects the retreat of ice from the Amundsen Sea Embayment in the HV Earth model run but not in the LVZ run), 1 m less for the 4 × CO_2_ scenario and 4.3 m less for the 8 × CO_2_ scenario.

To investigate the origin of these differences, we compare changes in bedrock elevation predicted using the HV and LVZ Earth models in the first 0.9 kyr of the 2 × CO_2_ simulation (Fig. 2b,c, respectively, see also Supplementary Fig. 2), just before the ice cover in the two simulations (and the rise in GSLE) begins to significantly diverge (green star in Fig. 1a). The ice thickness after 0.9 kyr in the HV Earth model run (which is nearly identical to the thickness in the LVZ run) is shown in Fig. 2a (left). Despite the similarity in ice cover, the change in elevation of the bedrock relative to the sea surface in the ice-sheet model (or equivalently, the negative of the change in sea level) is markedly different. The simulation adopting the thin lithosphere, low asthenospheric viscosity Earth model LVZ has experienced larger and more localized bedrock uplift (contributing to sea-level fall) in the vicinity of the grounding line than the simulation based on model HV (compare Fig. 2b,c). Indeed, the bedrock elevation near the grounding line in the latter simulation is up to 80 m lower than in the former. A thicker lithosphere acts to dampen and smooth the peak uplift in areas of ice loss. In contrast, the presence of a low-viscosity zone localizes deformation, and decreases the decay time of the uplift so that significantly more viscous deformation occurs in the first 0.9 kyr of the simulation. As a consequence, the sea-level stabilization associated with the LVZ model is more pronounced and the associated changes in GSLE in the two simulations begin to diverge. In fact, the uplift of the bedrock near the grounding line in the LVZ case is sufficient to initiate an advance of the ice sheet, and a drop in GSLE beginning ∼1.5 kyr into that simulation (Fig. 1a). Re-advance also occurs ∼2 kyr into the 4 × CO_2_ scenario for both Earth models (Fig. 1b).

In the 8 × CO_2_ emission scenario, the ice-sheet evolution in the HV and LVZ model simulations begin to diverge after 0.6 kyr (Fig. 2d–f, see also Supplementary Fig. 3). As in the 2 × CO_2_ case, the bedrock uplift (sea-level fall) is higher in amplitude and more localized near the grounding line in the LVZ model simulation relative to the HV case. However, in this case, the climate forcing is sufficiently strong that the ice volume plateaus after ∼1.5 kyr for both the LVZ and HV simulations rather than increasing (or, equivalently, the GSLE stays constant rather than falling beyond this point in the simulation). Note that in this high-emission scenario, significant retreat occurs in marine sectors of the EAIS, where the HV model may represent the Earth structure better than the LVZ model. We show the contributions from East and West Antarctica to the total Antarctic ice loss plotted in Fig. 1a,c in Supplementary Fig. 4.

### Impact of self-gravity on future ice loss

**Figure 3:**
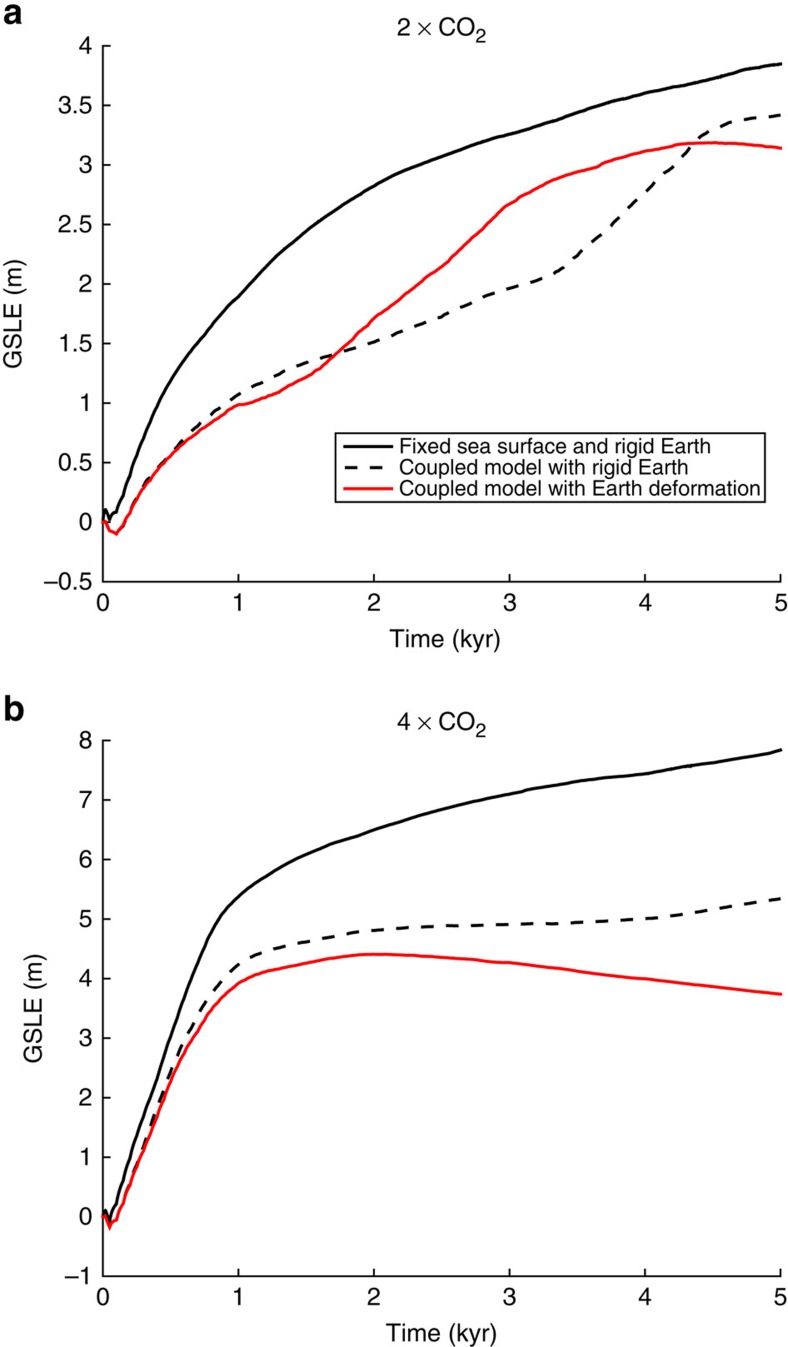
Relative impact of self-gravity and earth deformation on AIS evolution. (**a**,**b**) Changes in grounded ice cover, in units of metres of GSLE, as a function of time for simulations in which CO_2_ levels are increased instantaneously to 2 (**a**) or 4 (**b**) times modern values. Solid black lines correspond to a simulation in which the sea-level stabilization mechanism is not included and the sea surface and solid surface are fixed. Dashed black lines correspond to a simulation with the sea-level coupling included, but on a rigid Earth, so that only gravitational and rotational effects and no solid earth deformation are included. Red lines correspond to projections with the fully coupled model, including viscoelastic deformation of the HV Earth model.

The glacial isostatic adjustment model predicts changes in sea level associated with rotational, gravitational and Earth deformational effects. Self-gravitation in the (ice plus ocean) surface mass load and crustal deformation dominate the sea-level signal in the vicinity of an evolving ice sheet. These signals are coupled as self-gravitation influences the ocean load, which in turn drives crustal deformation. To investigate the importance of self-gravitation in our simulations, in Fig. 3, we compare results generated using the coupled ice sheet–sea-level model in a case where the solid Earth is rigid (that is, deformational effects are ignored; dashed black line) to earlier simulations in which the solid surface and sea surface are fixed (solid black line) or the full sea-level model that accounts for viscoelastic earth deformation is adopted (solid red line). We conclude that the loss of gravitational attraction between the melting ice sheet and ocean (and the associated subsidence of the sea surface near the grounding line) contributes significantly to the stabilizing influence of the sea-level feedback.

For the case of an instantaneous warming to four times preindustrial CO_2_ levels (3b), the model that incorporates self-gravitation in the surface mass load yields 60% of the grounded ice loss predicted in the simulation that includes full gravitational and deformational effects in the sea-level calculator. The relative importance of self-gravitation in these coupled ice sheet–sea-level simulations will depend on many factors, including the strength of the climate warming (for example, compare Fig. 3a and b), viscoelastic Earth structure, bedrock configuration, and rate and spatial pattern of ice loss. In addition, we note that the response of the system may depend strongly on initial conditions adopted in the simulations^5^,^24^. In any event, it is clear that gravitational effects on ocean mass redistribution should, in general, be considered in projections of ice-sheet stability.

### Sensitivity analysis

To this point, we have adopted a near-instantaneous ramp up of greenhouse forcing. We also ran a suite of simulations in which the duration of the linear increase in the forcing was set to 1 kyr (blue lines, Fig. 1a–c). In the case of the instantaneous warming scenario (red lines in Fig. 1a–c), most of the ice-sheet retreat takes place within the first 0.5 kyr (Fig. 1c) to 1 kyr (Fig. 1a) of the simulations, and this collapse is followed by a more gradual ongoing evolution of ice mass. When the greenhouse forcing is increased more gradually, over 1 kyr, the majority of the ice loss is delayed by ∼1 kyr, but the duration of the collapse phase is largely unaltered. (The phase of ice growth preceding the retreat in the 1 kyr climate ramp up (blue lines, Fig. 1a–c) is due to increased snowfall.)

**Figure 4:**
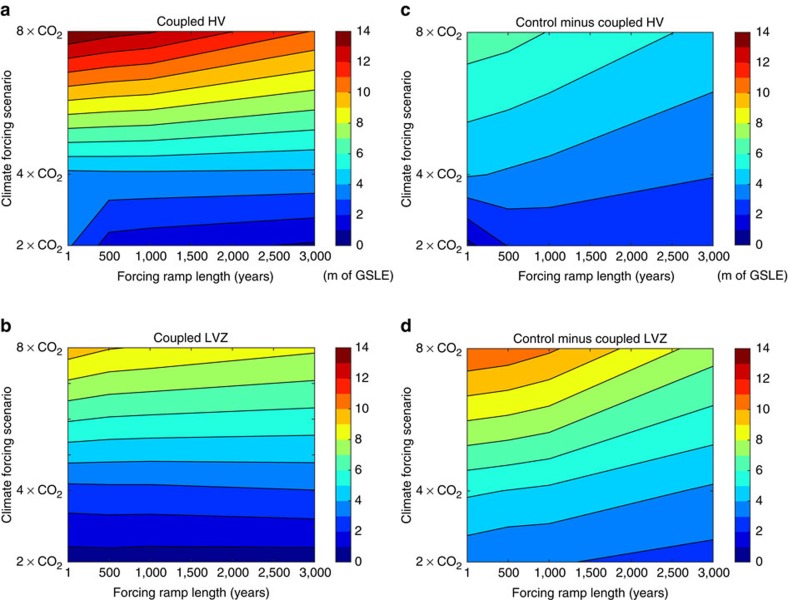
Sensitivity study for projections of Antarctic ice loss that vary the amplitude and timing of the imposed climate warming. (**a**,**b**) Total Antarctic ice loss, in metres of GSLE, for 5 kyr simulations with the HV (**a**) and LVZ (**b**) Earth models that vary the magnitude (*y* axis, 2, 4 or 8 × CO_2_) and timing (*x* axis, calculated for ramp lengths of 1 year (that is, instantaneous), 500 years, 1 kyr, 2 kyr and 3 kyr) of the greenhouse emission. (**c**,**d**) Difference in total ice loss in metres of GSLE between the control run that includes no bedrock or sea surface height changes and the coupled HV (**c**) and LVZ (**d**) Earth model simulations.

There are two timescales of importance to the ice-sheet collapse. The first is the timescale for the break up of ice shelves that are buttressing the grounded portion of the ice sheet; this timescale is a strong function of temperature, and therefore, the greenhouse emission scenario. The second is the timescale of ice-sheet collapse; this collapse is driven by the marine ice-sheet instability mechanism^25^,^26^, which depends primarily on geometry of the bed at the grounding line, and it is less sensitive to the level of climate and ocean warming^3^,^6^,^27^,^28^. To extend this analysis further, we performed a large suite of simulations in which the CO_2_ ramp-up time ranged from instantaneous to 3 kyr for each emission scenario and Earth model (Fig. 4 and Supplementary Fig. 5). In general, a larger climate forcing results in more ice loss, but the time over which the forcing is applied does not have a strong influence on the total ice loss at the end of the simulation (Fig. 4a,b). The sea-level feedback has greater impact on the amount of ice loss for larger climate forcing and to a lesser degree, for longer forcing timescales (Fig. 4c,d). Note that, as mentioned in the discussion of Fig. 1 above, these trends do not necessarily apply to the percent difference in ice loss between the coupled and control runs.

## Discussion

The fate of the polar ice sheets in a progressively warming world is a focus of climate research and a concern for policy makers and the general public. Using a coupled sea level–three-dimensional ice-sheet model, we have demonstrated that sea-level changes associated with uplift of the solid Earth in response to reduced ice and ocean loading, and draw down of the sea surface because of gravitational effects discussed previously in the context of the last ice age^11^,^12^,^13^, may have a significant impact on future AIS mass loss. The reduction in the predicted rise in GSLE will be a function of the climate forcing, with higher forcing associated with a smaller percentage drop in the GSLE rise (but a higher absolute decrease in GSLE) relative to simulations in which the stabilization is not included. Furthermore, the impact of the sea-level stabilization is also sensitive to the Earth model adopted to compute the load-induced deformation component of sea-level changes in the Antarctic region. In this regard, Earth models that are consistent with geological and seismological evidence for a thin lithosphere and low-viscosity zone beneath the WAIS predict less ice loss than Earth models that have been derived from analyses of globally distributed ice age data sets and are more representative of the structure beneath the EAIS. Future modelling developments to incorporate the strong lateral variations in viscoelastic structure beneath Antarctica and explore the impact of nonlinear Earth rheologies are clearly warranted^28^. Our simulations also show that bedrock elevation changes may be particularly critical to the future stability of grounded, marine-based sectors of ice in the Amundsen Sea Embayment, which are often identified as the strongest contenders for large-scale collapse in the coming centuries^2^,^3^,^5^,^29^, motivating the development of regional, high-resolution studies of ice sheet–sea level–solid Earth interactions. In general, we recommend that future ice model intercomparison studies in Antarctica (for example, ISMIP6 and MISOMIP ) should include a consideration of the impact of gravitational effects and variations in Earth structure.

## Methods

### Earth structure

We performed simulations with a suite of Earth models with distinct thicknesses of an elastic lithosphere and radial profiles of mantle viscosity. In the main text, we consider two models: model HV has a lithospheric thickness of 120 km, and upper and lower mantle viscosities of 5 × 10^20^ and 5 × 10^21^ Pa s, respectively (for example, see refs 18, 19 for details); model LVZ has a 50-km-thick lithosphere, a low viscosity of 10^19^ Pa s to a depth of 200 km, a viscosity of 2 × 10^20^ Pa s in the remaining upper mantle and 3.0 × 10^21^ Pa s in the lower mantle. This model is considered consistent with the geological setting of West Antarctica and seismic tomographic constraints on mantle structure^20^,^23^ (see main text).

In Supplementary Fig. 1, we consider coupled sea level–ice-sheet simulations for several other Earth models. Models 80 HV and 50 HV are identical to the HV model with the exception that the lithospheric thickness is reduced to 80 or 50 km, respectively. Models 50_p2_3 and 50 LVZ_p5_5 are variations on the model LVZ. The first model is the same as model LVZ, except the low-viscosity zone is replaced by the viscosity of the remaining upper mantle. The second changes the upper and lower mantle viscosities of the LVZ model to 5 × 10^20^ and 5 × 10^21^ Pa s, respectively.

All the models adopted in this study have a linear Maxwell viscoelastic rheology with radially varying Earth structure. In future work, we will investigate the impact of incorporating laterally varying Earth properties, and nonlinear rheologies into the coupled sea level–ice-sheet simulations.

### Modelling

To model AIS thickness and bedrock evolution together with global sea-level changes under a series of climate forcing scenarios, we employ the coupled ice sheet–sea-level model described in ref. 11 with forcing and initial bedrock configuration appropriate to modern (rather than ice age) conditions. The model couples the ice-sheet/shelf model of ref. 14 to a gravitationally self-consistent sea-level model that incorporates deformation of a rotating, viscoelastic Earth model with radially varying Earth structure and migrating shorelines, including the inundation of water into regions freed of marine ice. The ice-sheet model uses a spatial resolution of 20 km, and the sea-level model calculations are performed at spherical harmonic degree 512. Fields are passed between the ice-sheet and sea-level models every 25 years in the simulations. We defer to ref. 11 for the model details, and describe only the model setup specific to the future climate warming simulations considered here.

To begin the initialization process, the ice-sheet model alone with no sea-level coupling is spun up to a modern equilibrium state forced with climatological ALBMAP atmospheric conditions^30^ and WOA2009 ocean temperatures^31^. Bedrock beneath the ice sheet is fixed to Bedmap2 bedrock elevation^32^ during this first spin up. Next, to initialize the coupled model, for each adopted model of Earth structure, a 5-kyr control run of the coupled model with constant climate conditions is performed with the ice sheet starting in the aforementioned modern equilibrium state. Climate is kept constant in the control run to allow the coupled system (that is, ice, bedrock and sea surface height) to adjust to the adopted Earth structure and settle into a (new) equilibrated modern-like state (see Supplementary Fig. 6 for details). We neglect the impacts of ongoing glacial isostatic adjustment because of pre-modern ice cover changes on ice dynamics (for example, see ref. 33 for details) here, as they will be negligible on the timescales and for the scale of ice-sheet collapse considered in this study. As initial bedrock topography for the coupled model spin up, we use etopo2 (http://www.ngdc.noaa.gov/mgg/fliers/01mgg04.html) global topography combined with Bedmap2 bedrock elevation over Antarctica^32^.

Starting from the equilibrated state for each Earth model, we perform a suite of 5-kyr-long coupled model simulations, applying a simple, linear ramp up of climate forcing, varying the length of the ramp from 1 to 3 kyr. For the atmosphere, the warming is applied as an anomaly to modern climatology. The anomaly is the difference in modelled atmospheric temperature (additive) and precipitation (multiplicative) between a modern control simulation and simulations for fixed CO_2_ values of 2, 4 or 8 × preindustrial CO_2_ level (280 parts per million by volume (p.p.m.v.)). The RegCM3 RCM^34^ with some adaptations for polar regions is used over Antarctica, driven by the GENESIS v3 Global Climate Model (GCM) with a slab ocean^35^. Incorporating the effects of changes in ocean dynamics around Antarctic basins presents a much greater challenge, and is currently infeasible on the timescales needed for this study; for simplicity, a ramped 2 °C warming is applied uniformly to observed ocean climatological temperatures in all cases. The value of 2 °C is based on independent coupled GCM model transient simulations of future climate (for example, see ref. 36 for details). Note that although lapse-rate corrections are performed in the ice-sheet model at each grid point as the ice surface lowers or rises, our results do not currently include a fully interactive coupling between the ice sheet and the atmosphere. However, in Supplementary Fig. 1, we show the results of simulations with a basic consideration of the feedback between ice elevation and atmosphere in one asynchronous step. In this simulation, the ice-sheet model in the 8 × CO_2_ scenario is forced by a climate model simulation, in which marine sectors of the West Antarctic have been removed, so that the ice-sheet elevation used in the RCM better captures the ice elevations predicted in the ice-sheet model. In future work, we will use asynchronous or transient climate models and representative concentration pathway (RCP) scenarios^1^ and consider such feedbacks between atmosphere and ice-sheet models.

The ice-sheet model in this paper does not include the new mechanisms of hydrofracturing by surface melt and ice-cliff failure, recently proposed^37^ to produce East Antarctic retreat as implied by (albeit uncertain) geologic evidence of high sea-level stands in past warm periods. A future paper exploring these effects with the coupled Earth–ice model is planned, but the mechanisms are somewhat speculative, and their effect is basically to accelerate WAIS retreat and amplify EAIS retreat, and the basic findings of this paper regarding negative-feedback influences of Earth-gravitational interactions are not expected to change. We note that the warmest (8 × CO_2_) experiments here produce substantial EAIS margin retreat (Figs 1f, 2d and Supplementary Fig. 3), not dissimilar to that in ref. 37 with cooler climate, but here due largely to strong surface melting in terrestrial ablation zones. Finally, following future ice-sheet modelling developments, it will also be important to assess the relative importance of the sea-level feedback and other factors influencing future ice loss (for example, surface mass balance, numerics of the grounding line and basal melting). We note that current ice-sheet models are not able to capture all of these effects accurately.

### Code availability

As understanding and running the coupled ice sheet–sea-level model code requires substantive training, codes associated with this work have not been made publically available. The ice sheet model alone from ref. 14 with no sea-level coupling is available upon request. The corresponding author may be contacted for published results and inquiries about modelling details.

## Additional information

**How to cite this article:** Gomez, N. *et al.* Sea-level feedback lowers projections of future Antarctic ice sheet mass loss. *Nat. Commun.* 6:8798 doi: 10.1038/ncomms9798 (2015).

## Supplementary Material

Supplementary Information Supplementary Figures 1-6
