# Supplementary material for: Sea-level feedback lowers projections of future Antarctic Ice-Sheet mass loss
Source: Nat Commun. 2015 Nov 10;6:8798. doi: 10.1038/ncomms9798 (PMC5426515; doi:10.1038/ncomms9798)
Supplement: Supplementary Information — Supplementary Figures 1-6 [file ncomms9798-s1.pdf]

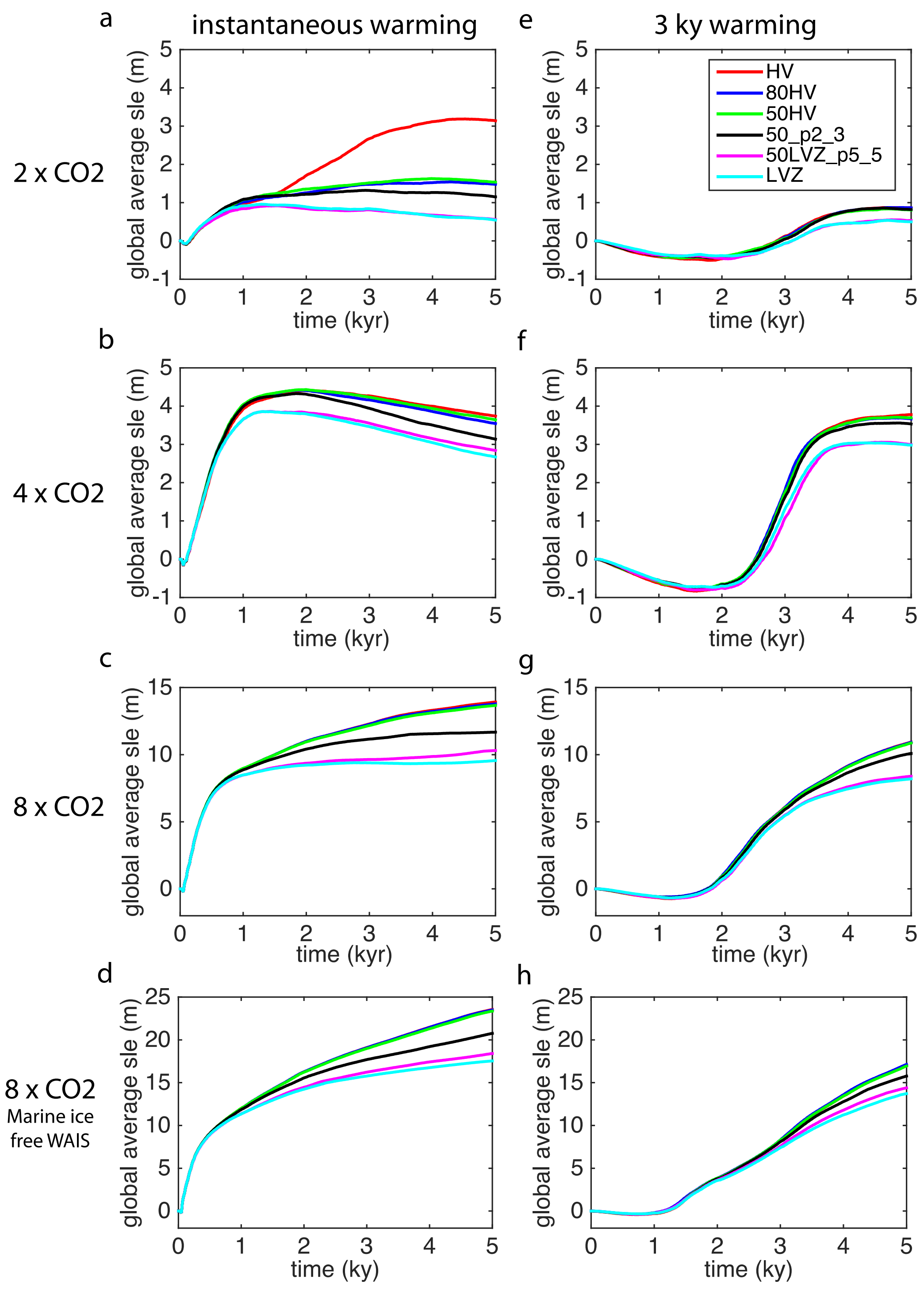

Supplementary Figure 1: Ice sheet retreat in simulations including the sea-level stabilization with a suite of different Earth models varying lithospheric thickness and mantle viscosity. (a-h) Changes in grounded ice thickness, in units of meters of GSLE, as a function of time for the Earth models indicated in the legend in frame (e) and described in the Methods section of the main text. Results are presented for an instantaneous increase in CO_2 emissions to 2 (a), 4 (b) and 8 (c) times their modern levels.. (e-g) As in (a-c) but for a 3 kyr linear ramp in CO_2 emissions, the maximum length of ramp considered in this study. (d) and (h) are as in (c) and (g) except that the ice sheet model is forced by a climate model simulation in which West Antarctic marine ice is replaced by ocean.

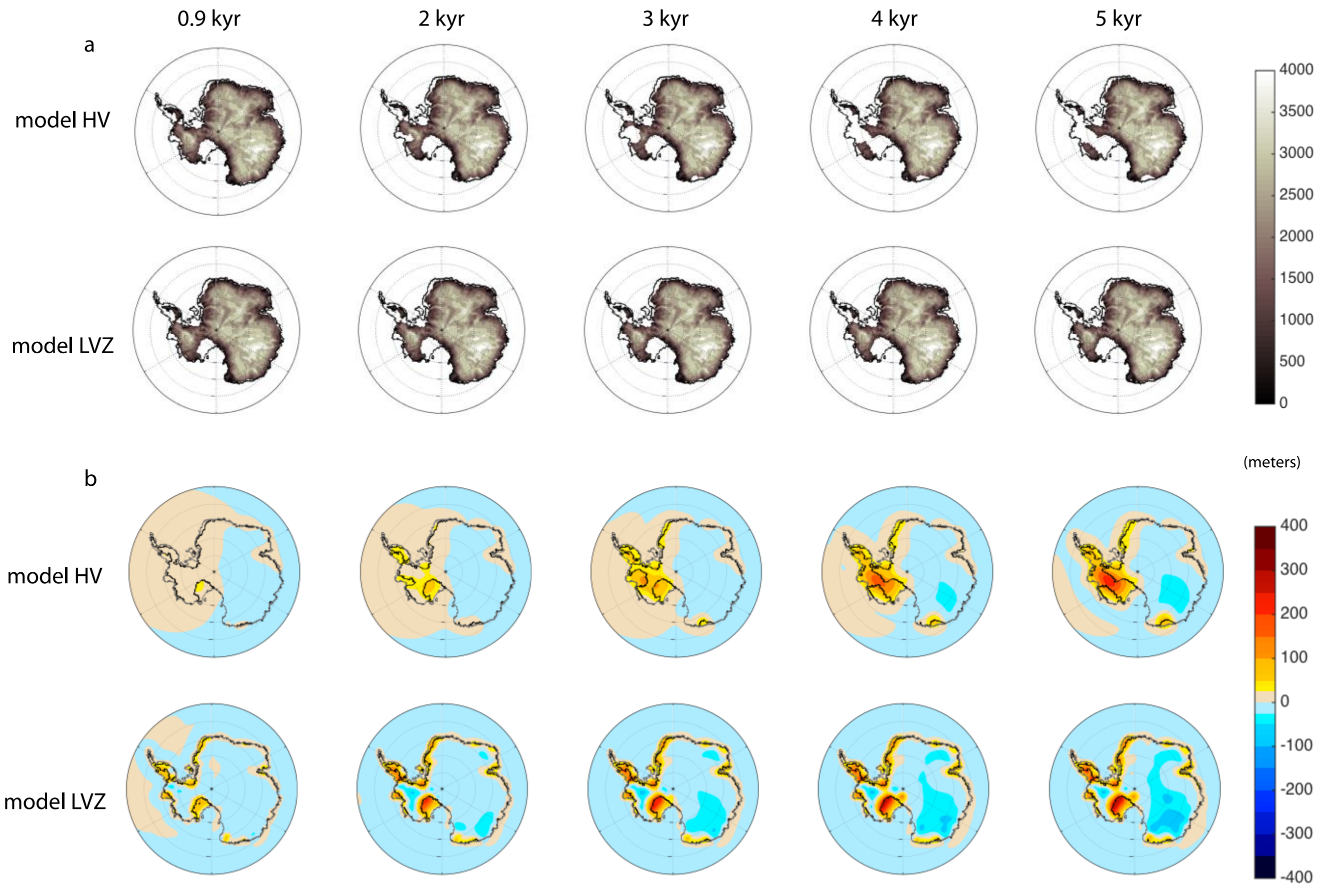

Supplementary Figure 2: Ice sheet and topography evolution predicted by the coupled model for the 1yr, 2xCO_2 emissions scenario. (a) Snapshots of grounded ice thickness in meters in Antarctica predicted using the HV (top row) and LVZ (bottom row) Earth model simulations at the times indicated at the top of the figure. Black lines indicate the grounding line position at the start of the model run. (b) Change in bedrock elevation in meters from the start of the simulation to the times indicated at the top of the figure, using the HV and LVZ Earth models (top and bottom rows, respectively, as in (a)). Black lines show the grounding line at the times indicated above, and grey lines show the grounding line at the start of the model run (0 kyr).

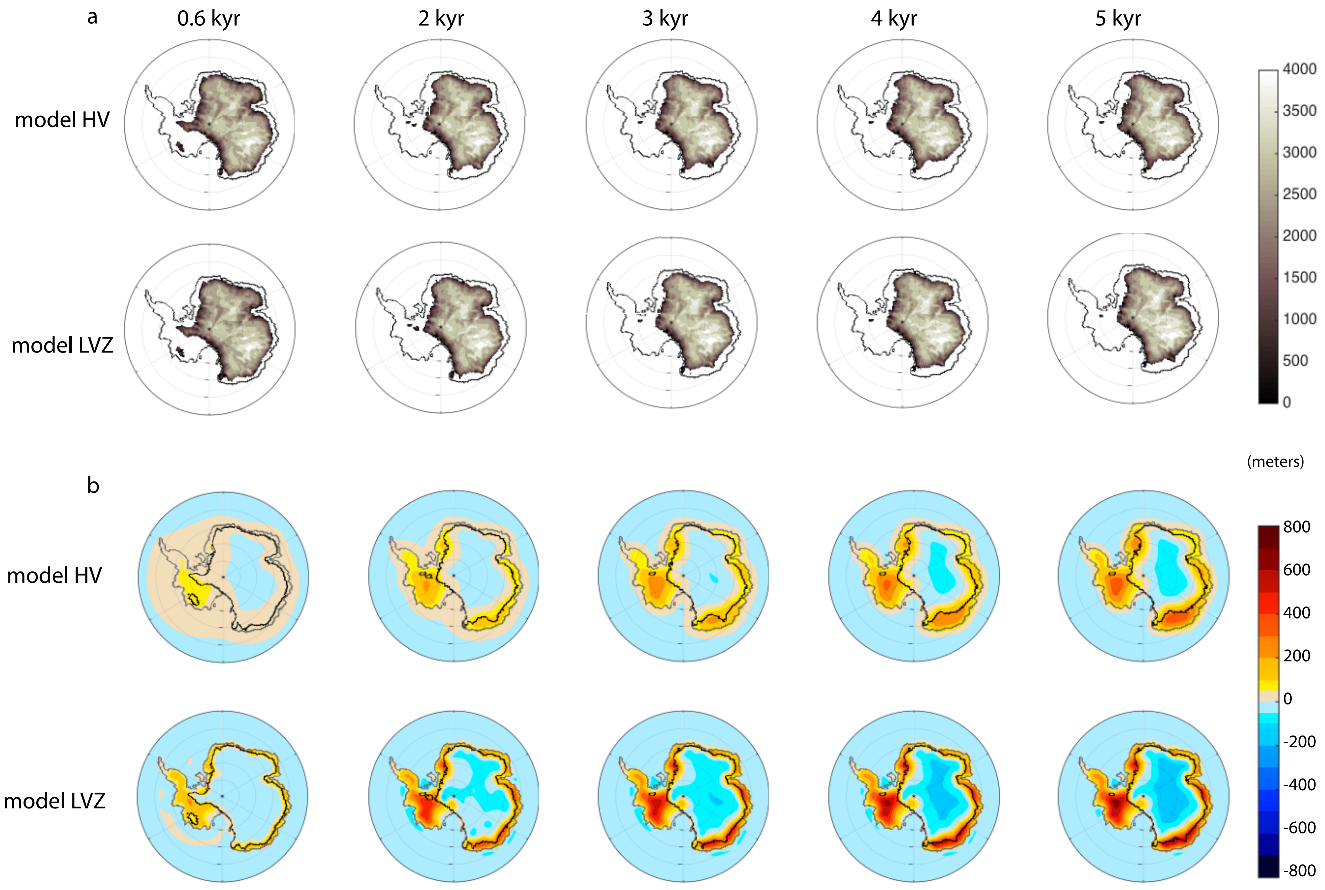

Supplementary Figure 3: Ice sheet and topography evolution predicted by the coupled model for the 1yr, 8xCO_2 emissions scenario. Frames are as in Supplementary Figure 2, but note the different colorbar in frame (b).

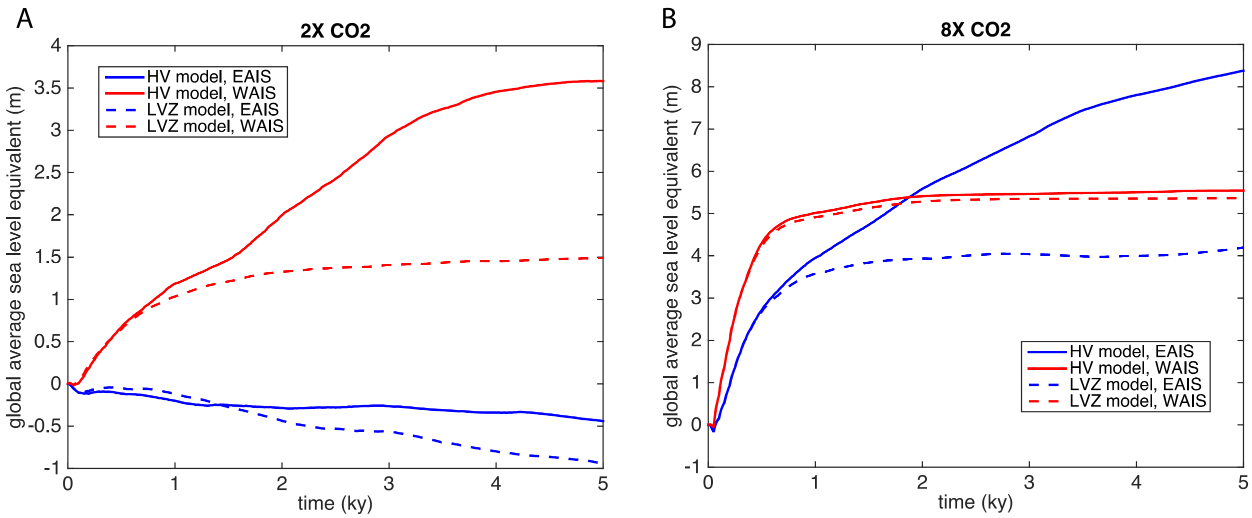

Supplementary Figure 4: Contributions of East and West Antarctica to total ice loss. Changes in grounded ice cover, in units of meters of GSLE, as a function of time for simulations in which CO_2 levels are increased to 2 (a) and 8 (b) times modern levels instantaneously. Projections based on the HV and LVZ Earth models are given by solid and dotted lines, respectively. Blue lines show the contribution from East Antarctic Ice Sheet (EAIS) and red lines show the contribution from the West Antarctic Ice Sheet (WAIS).

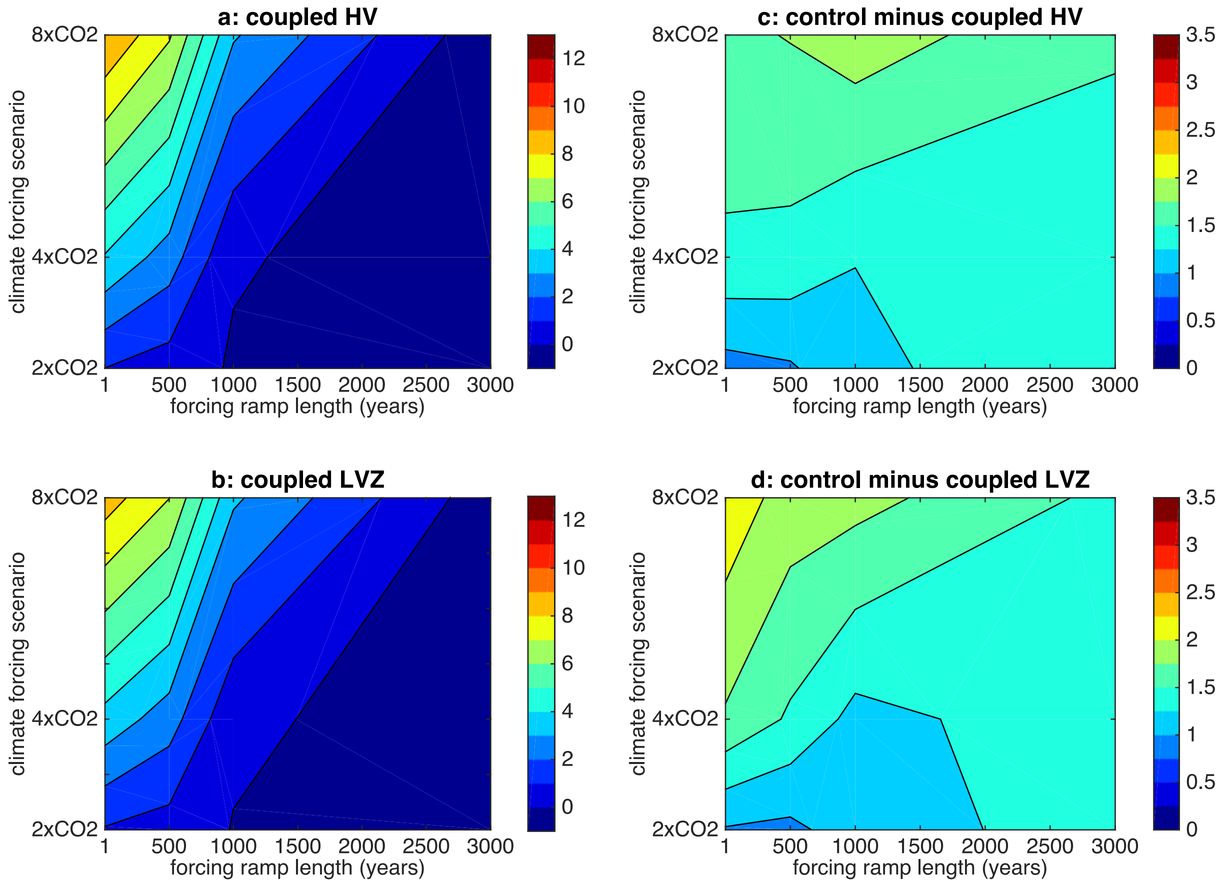

Supplementary Figure 5: Sensitivity study for predictions of AIS melt loss over 1000 years that vary the amplitude and timing of the imposed climate warming. As in Figure 3 of the main text, but focusing on the first 1000 years of the simulations. (a-b) Total AIS mass loss after 1000 years, in meters of GSLE, with the HV (a) and LVZ (b) Earth models that vary the magnitude (y-axis, 2, 4 or 8 × CO_2) and timing (x-axis, calculated for ramp lengths of 1 yr (i.e. 'instantaneous'), 500 yrs, 1 kyr, 2 kyr and 3 kyr) of the greenhouse emission. (c-d) difference in total mass loss after 1000 years in meters of GSLE between the control run that includes no bedrock or sea surface height changes and the coupled HV (c) and LVZ (d) Earth model simulations.

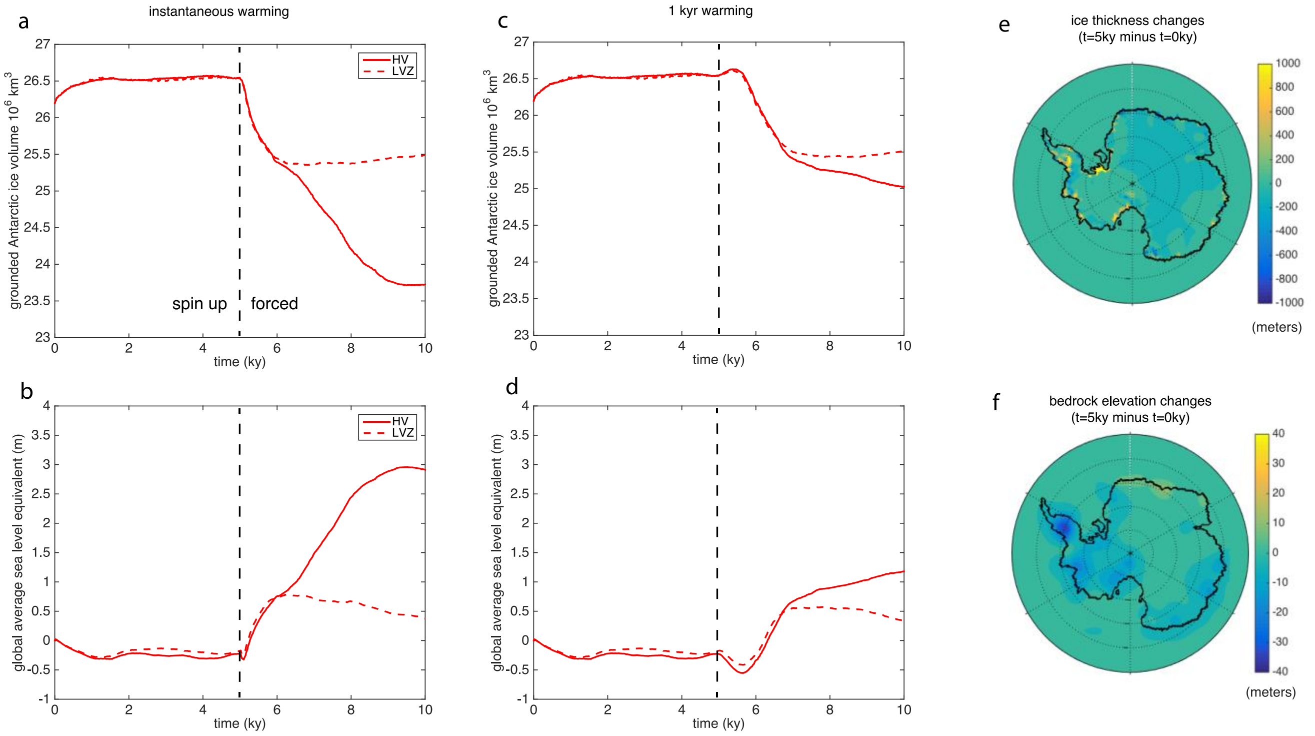

Supplementary Figure 6: Ice sheet evolution during and after the initialization of the coupled model to modern conditions. Antarctic ice sheet volume (a,c) and GSLE (b,d) as a function of time are plotted with the HV and LVZ Earth model for the instantaneous (a-b) and gradual 1000 year (c-d) warming scenarios. 0 ky on the x axis corresponds to the start of the unforced, initialization of the coupled model, and climate warming is introduced at 5ky, indicated by the vertical black dashed lines. (e-f) changes in ice thickness (e) and bedrock elevation relative to the sea surface (f) in meters in the HV Earth model simulation from the start of the initialization (0ky in frames a-d) to the time when the forcing is applied (5ky in frames a-d).
